# Glycyl tRNA Synthetase (*GARS*) Gene Variant Causes Distal Hereditary Motor Neuropathy V

**DOI:** 10.1155/2018/8516285

**Published:** 2018-01-30

**Authors:** Peter Chung, Hope Northrup, Misbah Azmath, Ricardo A. Mosquera, Shade Moody, Aravind Yadav

**Affiliations:** ^1^Children's Memorial Hermann Hospital, Houston, TX, USA; ^2^Department of Pediatrics, McGovern Medical School, University of Texas Health Science Center, Houston, TX, USA

## Abstract

Distal hereditary motor neuropathies (dHMN) are a rare heterogeneous group of inherited disorders specifically affecting the motor axons, leading to distal limb neurogenic muscular atrophy. The *GARS* gene has been identified as a causative gene responsible for clinical features of dHMN type V in families from different ethnic origins and backgrounds. We present the first cohort of family members of Nigerian descent with a novel heterozygous p.L272R variant on the *GARS* gene. We postulate that this variant is the cause of dHMN-V in this family, leading to variable phenotypical expressions that are earlier than reported in previous cases. The exact cause for the observed clinical heterogeneity within the family is unknown. One explanation is that there are modifier genes that affect the phenotype. These cases highlight the possibility of considering pathogenic variants in the *GARS* gene as a potential cause of early onset axonal polyneuropathy with atypical presentation.

## 1. Introduction

Distal hereditary motor neuropathies (dHMN) are a rare heterogeneous group of inherited disorders specifically affecting the motor axons, leading to distal limb neurogenic muscular atrophy. dHMN are classified into seven subtypes based on clinical and genetic features [1]. Different causative genes provide part of the basis for the observed clinical heterogeneity of dHMN disease. Interestingly, amongst the seven genes that have been identified to cause the disease, most have housekeeping functions, including small heat shock proteins (*sHSP*) (dHMN-II); glycyl tRNA synthetase (*GARS*) (dHMN-V); Berardinelli–Seip congenital lipodystrophy (*BSCL2*) (dHMN-V); immunoglobulin *µ*-binding protein-2 (*IGHMBP2*) (dHMN-VI); dynactin (*DCTN1*) (dHMN-VII); and senataxin (*SETX*) (dHMN) with pyramidal tract signs.

Of these, the *GARS* gene located on chromosome 7p produces an aminoacyl tRNA synthetase responsible for charging the associated tRNAs and the first protein of its kind implicated in any inherited human disorder. Two transcript variants encoding different isoforms have been found for the gene, leading to dHMN-V with a pure motor form and Charcot-Marie-Tooth 2D (CMT2D) with both motor and sensory involvement [2]. dHMN-V is a rare subtype inherited in an autosomal dominant manner, characterized by a preferential involvement of the upper limb muscles. It causes distal weakness and wasting that typically manifests later in life around adolescence, with onset varying from infancy to the third decade of life. To date, the exact mechanism of how pathogenic variants in the *GARS* gene cause such a specific neuropathy remains unknown.

Here, we present the first dHMN-V reported case of Nigerian descent with a novel variant of unknown significance (VUS) on the *GARS* gene that we postulate results in atypical and heterogeneous phenotypes within a single family.

## 2. Clinical Presentations

Sibling #1 is a 2-year-old female, born at full-term, who presented with acute respiratory failure at 10 months of age. She was found unresponsive for an unknown time, needing CPR and emergent intubation. Laboratory investigation was significant for positive RSV and adenovirus coinfection on nasal swab. Two days later, she was extubated and placed on nasal cannula oxygen therapy; however, physical examination showed hypotonia and hyperlaxity of her extremities which prompted further neurological evaluation. On EMG, there was no sensory function abnormality, but motor axonopathy concerning for underlying neuromuscular disorder was detected. Two months later, she was readmitted for respiratory failure as well as failure to thrive due to poor feeding. Her poor feeding was thought to be secondary to underlying neuromuscular disorder, but no etiology was found for her respiratory failure this time. She ultimately underwent G-tube with fundoplication and was discharged home on continuous positive airway pressure. One month later or three months after her initial presentation, she was admitted again for respiratory failure that required intubation. Due to chronic, multiple respiratory failure and inability to extubate and transition to noninvasive ventilation, she underwent tracheostomy for long-term invasive ventilation at 13 months of age. Muscle biopsy results performed at this time were consistent with infantile denervation as seen with anterior horn cell disease in spinal muscular atrophy or motor involvement in a motor-sensory neuropathy. Testing for mitochondrial disease including standard pathological testing of muscle biopsy as well as qPCR analysis for mitochondrial DNA content and respiratory chain enzyme analysis (ETC) of skeletal muscle was negative. Samples were sent for whole exome sequencing (WES) which then revealed a variant of unknown significance (VUS) with a heterozygous c.815T > G (p.L272R) in the *GARS* gene. Currently, she is able to sit unsupported but is unable to walk and is wheelchair bound.

Sibling #2 is a 4-year-old female, born at full-term, with no significant past medical history, who developed appropriate gross motor milestones until 9 months of age. She then became delayed experiencing hypotonia and hyperlaxity with predominant weakness and muscle wasting in her upper and lower distal extremities. Despite physical, occupational, and speech therapy, her motor skills regressed, requiring orthotics to assist with motor functions.

EMG was also performed, which showed normal sensory function with no myopathic process; thus, no clear diagnosis was established. Unlike her sister, she does not have gastrointestinal or respiratory symptoms. She has adequate airway clearance with strong cough and clearing of her secretions. Further pulmonary workup including blood gas and a polysomnography did not show evidence of hypoventilation syndrome secondary to her underlying neuromuscular disorder. She too tested positive for the novel heterozygous missense sequence variant in exon 7 of the *GARS* gene, c.815T > G (p.L272R), interpreted as variant of unknown significance.

Sibling #3 is a 7-month-old male, born at full-term, otherwise healthy with no significant past medical history, who presented with acute hypercapnic respiratory failure. The patient had decreased activity level and oral intake prior to presentation. Physical exam was significant for grunting, tachypnea, subcostal retraction, and decreased breath sounds on the left lung field as well as hypoxia. Laboratory tests were normal except for an abnormal arterial blood gas, indicating acute respiratory failure that led to the use of continuous positive airway pressure followed by endotracheal intubation. Chest x-ray revealed infiltrates suggestive of left lower lobe pneumonia, subsequently treated with antibiotic therapy. The use of respiratory support was difficult to de-escalate as the patient failed multiple extubation attempts. A bronchoscopic evaluation did not reveal any airway anomalies. Underlying neuromuscular disorder was suspected due to family history of prior similar presentation. After several weeks, the patient was successfully extubated to bilevel positive airway pressure before being discharged home on noninvasive ventilation. Due to his ongoing need for respiratory support, he subsequently underwent tracheostomy. His physical examination is significant for hypotonia and hyperlaxity of extremities consistent with the siblings. Presence of heterozygous c.815T > G (p.L272R) variant of unknown significance (VUS) in the glycyl tRNA synthetase (*GARS*) gene was also confirmed. Findings of the family are summarized in Table 1.

Both parents are asymptomatic and without medical condition. Upon whole exome sequencing (WES) of the parents, the 37-year-old father of the 3 siblings did not have any abnormalities detected. The 36-year-old healthy mother was mosaic for the same *GARS* variant at a penetrance level of 10–20%. The variant has never previously been described; and not found in the EVS AA/EA databases, and predicted to be damaging (SIFT) and probably damaging (PolyPhen-2). All other variants of unknown significance detected on WES resulting in autosomal dominant conditions were inherited from one of the asymptomatic parents.

Otherwise, there is no history of consanguinity, and there were no other family members with birth defects, learning disabilities, early deaths, pregnancy wastage, and similar symptoms to our patients.

## 3. Discussion

Pathogenic variants in the glycyl tRNA synthetase (*GARS*) gene have been described in association with a specific form of an autosomal dominant motor axonal neuropathy that preferentially involves the upper limbs known as distal hereditary motor neuropathy V (dHMN-V) or spinal muscular atrophy type Va (dSMA-Va). Discovered in 2003, this is the first example of an aminoacyl tRNA synthetase being implicated in any inherited human disorder, and so far 11 pathogenic missense *GARS* variants have been linked to not only dSMA-V but also Charcot-Marie-Tooth disease type 2D (CMT2D) [2]. These variants in the *GARS* gene have been described in families from different ethnic origins and backgrounds including Bulgarian, North American, Mongolian, Algerian Sephardic Jewish, French, British, Italian, Australian, Ghanan, Japanese, and Korean [3–5]. Here, we present the first family of Nigerian descent with a novel missense variant in the *GARS* gene. A thorough evaluation failed to reveal any other cause for the phenotypes observed in the reported family. Furthermore, two of the three affected siblings presented with a severe phenotype including infantile onset respiratory distress and proximal muscle involvement.

Pathogenic variants in the *GARS* gene are associated with two allelic subtypes: Charcot-Marie-Tooth Type 2D (CMT2D) and distal hereditary motor neuropathy type V (dHMN-V) also known as spinal muscular atrophy type Va (dSMA-Va). The distinction between CMT2D and dHMN-V is based on phenotypical presentation of sensory deficits in the form of reduction in temperature, touch, pinprick, and vibration perception in a glove and stocking pattern present in the latter [6]. Patients with pathogenic variants in the *GARS* gene have typically presented with progressive bilateral weakness, vibratory sense impairment in extremities, and hand muscles atrophy, specifically the thenar and first dorsal interosseus muscles with relative sparing of the hypothenar eminence. Subsequent involvement of the lower limb muscles has been noted in less than half of described patients. Although it is difficult to determine with a high degree of certainty the presence of any sensory abnormalities among our patients due to a very early onset of symptoms, their motor presentations are consistent with dHMN-V.

The diagnosis of *GARS*-associated axonal neuropathy is made based on phenotypical presentation, electromyography (EMG), and genetic testing. Early signs of clinical symptoms may include transient muscle cramping or pain of the extremities after cold exposure or exertion. Although the onset of symptoms differs among individuals and families, many people typically begin experiencing symptoms during adolescence or young adulthood. Onset of reported cases ranges mostly from second to fourth decades of life. Along with a previously reported infantile presentation in monozygotic twins, these siblings' presentations are one of the earliest onset of dHMN-V ever reported associated with a *GARS* variant. Previous cases of early infantile onset report *GARS* variant in the anticodon binding domain [7]; however, our siblings had GARS variant outside this domain. A heterozygous change in the same amino acid, reported as c.815T > A, p.L218Q has been previously reported in a patient with motor-dominant neuropathy with respiratory failure but of later onset [8]. Progression of the disease in the two youngest siblings is more rapid than in previous reports. Furthermore, their presentations are atypical in that proximal muscles are affected significantly more, leading to respiratory failure, rather than typical distal motor neuropathy. In contrast, the oldest sibling's phenotype is less pronounced with no respiratory failure. Her symptoms involve mostly the limbs and subsequent decline in gross motor functions, requiring prosthetic or assistive devices. Nonetheless, the age of onset in the oldest sibling is still early compared to the mean age of onset reported in the current literature. The heterogeneous phenotype pattern remains uncertain.

While the *GARS* gene is one of 37 tRNA synthetase genes ubiquitously expressed and absolutely necessary for protein synthesis in all cells, the exact mechanism of how pathogenic variants in the gene can result in a very specific form of neuropathy remains elusive. In fact, since its identification, four other tRNA synthetase genes have been linked to neurological phenotypes in humans or mice, thus indicating the important role they play in the development and maintenance of the nervous system. Although it is conceivable that neurons may be more prone to a loss or decrease in tRNA synthetase, it has been suggested that *GARS* could have an alternate function specific to neurons or interact with an unknown molecular partner in the peripheral nerve via a gain of function mechanism.

Our patients, despite sharing the same genetic variant, have varying degree of onset and severity in their phenotypical presentation. For example, two siblings have respiratory failure but at different ages and varying severity, while one sibling does not have any respiratory issue. Such a phenomenon may further suggest the role of an unknown molecular partner or modifier that secondarily affects these phenotypes. Furthermore, the severe phenotypic presentation in our patients stresses the important role *GARS* plays in the peripheral nervous system. Genetic studies revealed that these children have inherited the variants from their mother, who is mosaic for the variant, explaining why she remains asymptomatic.

Currently, treatment and prognosis remain unclear for the affected children. There have been recent reports of novel de novo pathogenic variants in the *GARS* gene leading to a severe presentation of HMN with onset during infancy resulting in permanent disability in the affected individuals around the age of 7–11 years [9, 10]. We suspect that their care will largely continue to remain supportive. In this family, since *GARS*-associated axonal neuropathy is autosomal dominant, there is up to 50% recurrence risk for every future pregnancy.

## 4. Conclusion

In summary, we postulate that the novel heterozygous p.L272R variant on the *GARS* gene described here is the cause of dHMN type V in this family of Nigerian descent. Interestingly, these sibling patients with the same genetic variant presented in an atypical manner with variable phenotypes. The exact cause for the observed clinical heterogeneity is unknown. One explanation is that there are modifier genes that affect the phenotype. With continuing advancements in genomic testing as well as more awareness and discovery of various genetic variants, we anticipate more insight into the *GARS* gene mutation and its role in axonal neuropathy, specifically dHMN-V. These cases highlight the possibility of considering pathogenic variants in the *GARS* gene as a potential cause of early onset axonal polyneuropathy.

## Figures and Tables

**Table 1:** Features and characteristics of patients' presentation.

	Patient #1	Patient #2	Patient #3	Mother
Phenotype	dSMA-V	dSMA-V	dSMA-V	—
GARS mutation	p.Leu272Arg variant	p.Leu272Arg variant	p.Leu272Arg variant	p.Leu272Arg variant
Age, current	2 years old	4 years old	7 months old	36 years old
Age of onset	10 months	9 months	7 months old	—
Axial and truncal (proximal) weakness	+	+	+	−
Hands/feet extremities (distal) weakness	+	+	+	−
Hypotonia	+	+	+	−
Hyperlaxity	+	+	+	−
Hyporeflexia	+	+	+	−
Respiratory failure	+	−	+	−
Cardiomyopathy	−	−	−	−

dSMA-V: distal spinal muscular atrophy type V; Leu: leucine; Arg: arginine; + present; − absent.

## Abbreviations

BSCL2: Berardinelli–Seip congenital lipodystrophy
CMT2D: Charcot-Marie-Tooth 2D
DCTN1: Dynactin subunit 1
dHMN: Distal hereditary motor neuropathies
dSMA-V: Distal spinal muscular atrophy type V
dSMA-Va: Distal spinal muscular atrophy type Va
EMG: Electromyography
ETC: Mitochondrial respiratory chain enzyme analysis
GARS: Glycyl tRNA synthetase
IGHMBP2: Immunoglobulin *µ*-binding protein-2
L: Leucine
R: Arginine
SETX: Senataxin
sHSP: Small heat shock proteins
SMA: Spinal muscular atrophy
RSV: Respiratory syncytial virus
VUS: Variant of unknown significance
WES: Whole exome sequencing.
